# Advice from the Health Ministry

**Published:** 1920-10-30

**Authors:** 


### MILK AND ITS GENERAL USES.

### Advice from the Health Ministry.

A circular accompanied by a pamphlet* on the milk question has just been issued to the local authorities by the Ministry of Health, and although, as has often been explained in these columns, the clean milk campaign has already made considerable progress through the excellent work of the National Clean Milk Society, yet the Ministry has wisely considered it advisable to place the official position more clearly before their local representatives.

In brief, the position at present stands that provision was made in the Milk and Dairies (Consolidation) Act of 1915 for securing a more adequate supervision of the conditions under which milk is produced, handled, and distributed, and that at present a Bill is before Parliament to amend that Act slightly before it comes into operation. *Inter alia*, this Amending Bill provides for the extension of the system of classification of milk contemplated in the original Act. There are already arrangements by which the Food Controller issues licences permitting and regulating the use of the terms "Grade A (certified) milk" and "Grade A milk" in connection with milk produced and handled in accordance with certain prescribed conditions. Apparently a criticism of the situation of the moment is that relatively little advantage has been taken of these seemingly excellent arrangements, and the Ministry is justifiably anxious that increased publicity should be given to their provisions.

To make the true importance of this publicity clearer, let us summarise the conditions under which high-grade milk can be thus certified. "Grade A milk" must be produced under specially clean hygienic conditions from a herd free from tuberculosis. A licence may be held to sell milk under this designation if, during that time, the herd contains no bovine which has not passed the recognised tubercular tests, and testing must be repeated at intervals, while the herds must be subjected to periodical veterinary examinations at intervals of not less than six months, a report being sent on each occasion to the Ministry of Food. Every facility must be afforded by the producer, and indeed by all who handle this milk before it reaches the consumer, for the taking of samples. The actual methods of production, dairy conditions, etc., must be of a sufficient standard to satisfy at any time the representative of the Ministry of Health, and the milk itself must be cooled on the farm, sent out in sealed containers, and must bear full description of the place, day, and time of production. Upon the retailers are imposed the following conditions:—

(1) The milk must be dealt with on premises, and by equipment and methods which, after inspection, have been certified as satisfactory.

(2) It must be sold by such dealers in the vessels in which they have received it, the seal being unbroken.

(3) Or it must be delivered to the consumer in bottles which have been previously sterilised by steam, filled on the premises of the retailer, and closed by discs or caps bearing the designation "Grade A milk," and the day of production.

* *Pamphlet in Regard to the Use of Milk.* H.M. Stationery Office, October 1920.

It will be at once apparent that a highly satisfactory milk can be in this way assured, and where such conditions are complied with, it is but a step further in refinement to produce Grade A (certified) milk, which in abstract requires the following:—

(1) The milk must be cooled and bottled immediately after production.

(2) Every bottle must be covered by a suitable cap overlapping the lip of the bottle, and this cap must bear the name and address of the producer and date.

(3) The milk must not at any time before delivery to the customer contain the *bacillus coli* in 0.1 c.c. or more than 30,000 bacteria per c.c.

(4) Under this title the milk must not be delivered more than two days after its actual production.

Farmers and others who supply these milks must keep accurate and separate accounts and records for the two grades, and this in such a way that inspection and checking are at any time possible.

It must be admitted that widespread introduction of this method, carrying as it does considerable administrative activities, will take some time before it is firmly established. But there is no doubt about its potential value. Dr. Addison strongly urges local authorities to encourage a growing public demand for such reliable milk, which is inevitably the most satisfactory way of bringing the producers to fall in with a plan they may otherwise find unprofitable. Also he considers it desirable that local authorities who undertake the provision of milk to mothers and children should endeavour to arrange that their source of supply is making use of the above facilities. The official note actually states, "The Ministry would be willing to sanction the distribution by local authorities of high-grade milk to expectant and nursing mothers and children under five in accordance with a scheme approved by them under the Maternity and Child-Welfare Act, and to pay grant in respect of the expenditure so incurred."

There appears, therefore, no lack of official encouragement towards the final success of the clean milk campaign among the masses. The pamphlet, to which we have already referred, will undoubtedly be of the greatest value to every health worker in presenting the essential facts in a convenient and more or less popular form to the public. A house-to-house distribution is not suggested, but the sketch should serve as an excellent basis for advice and oral and practical instruction of the people. It will be sufficient in our present note to mention only one or two outstanding points concerning milk as a food, for we have repeatedly urged the broader truths, and that over a period of many years.

The vitamine content of milk is now an accepted and widely known fact. Yet it is still said by many of the working classes that milk as a food is too dear. But milk for the children is not dear even

**Milk and its General Uses**—(*continued*).

to-day, if it be compared with other foods. It is estimated that a quart of milk is equal to almost a pound of lean beef, or to ten eggs, or to three and three-quarter pounds of fresh fish.

Medical opinion holds that children under eighteen months of age require one and a half pints of milk a day after they are weaned, and older children one pint. Milk is wholesome for everyone, but those who need it most are expectant and nursing mothers, children, infants, and little children. Between them these classes form no inconsiderable proportion of the population. Yet the average consumption of milk for the whole of Great Britain is only about a quarter of a pint per head per day. Admitting that in some residential districts the figure is much higher than this, we also know that in many industrial quarters it is decidedly lower—in some cases as little as one-tenth of a pint per head. Evidently a great many people are not getting as much milk as they ought, whatever its quality may be.

At the same time there is the other side of the milk question—namely, cleanliness. Considerable amounts of dirt, quite other than bacteria, may get into milk, but not without the companionship of the latter. The ease with which disease germs gain access, the dangers of infantile diarrhœa, tuberculosis, typhoid, scarlet fever, diphtheria, etc., in their relation to milk can never be too insistently explained to the public; but here we are coming to facts we have been many times at pains to recount. So have many thousands of health workers all over the country. And still there is untold work to be done before the educational campaign is even partially complete. It is more than satisfactory that the Ministry is keenly alive to the value of personal instruction of the people, as preceding administrative measures, which half understood would inevitably be half wasted also.

